# miR-1207-5p suppresses lung cancer growth and metastasis by targeting CSF1

**DOI:** 10.18632/oncotarget.8718

**Published:** 2016-04-13

**Authors:** Wei Dang, Zailong Qin, Songqing Fan, Qiuyuan Wen, Yuanjun Lu, Jia Wang, Xuemei Zhang, Lingyu Wei, Wei He, Qiurong Ye, Qun Yan, Guiyuan Li, Jian Ma

**Affiliations:** ^1^ Hunan Cancer Hospital and the Affiliated Cancer Hospital of Xiangya School of Medicine, Central South University, Changsha, Hunan, China; ^2^ Cancer Research Institute, Central South University, Changsha, Hunan, China; ^3^ Hunan Key Laboratory of Nonresolving Inflammation and Cancer, Key Laboratory of Carcinogenesis of Ministry of Health, Key Laboratory of Carcinogenesis and Cancer Invasion of Ministry of Education, Changsha, Hunan, China; ^4^ Department of Pathology, The Second Xiangya Hospital of Central South University, Changsha, Hunan, China; ^5^ Xi'an Children's Hospital, Xi'an, Shaanxi, China; ^6^ Department of Clinical Laboratory, Xiangya Hospital of Central South University, Changsha, Hunan, China

**Keywords:** miR-1207-5p, CSF1, macrophage, tumor microenvironment, lung cancer

## Abstract

We previously reported that miR-1207-5p can inhibit epithelial-mesenchymal transition (EMT) induced by growth factors such as EGF and TGF-β, but the exact mechanism is unclear. Here we identified that Colony stimulating factor 1 (*CSF1*) is a target gene of miR-1207-5p. CSF1 controls the production, differentiation and function of macrophage and promotes the release of proinflammatory chemokines. We showed that miR-1207-5p inhibited lung cancer cell A549 proliferation, migration and invasion *in vitro*, and suppressed the STAT3 and AKT signalings. miR-1207-5p overexpression can increase HUVEC angiogenesis, and can modulate the M2 phenotype of macrophage. miR-1207-5p also significantly inhibited A549 cells metastasis in a nude mouse xenograft model. miR-1207-5p and CSF1 expression levels and their relationship with lung cancer survival and metastasis status were assayed by means of a lung cancer tissue microarray. Macrophage is an essential part of the tumor microenvironment, thus the miR-1207-5p-CSF1 axis maybe a new regulator of lung cancer development through modulating the tumor microenvironment.

## INTRODUCTION

We previously identified three microRNAs (miR- 148a, miR-505 and miR-1207-5p) that provide feedback regulation of epithelial-mesenchymal transition (EMT) induced by growth factors such as EGF and TGF-β. MicroRNAs (miRNAs) confer robustness to biological processes by targeting key players that are involved in previous development stages. We reported that miR-1207- 5p acted as a new negative regulator of EMT, suppressing cancer cell invasion and metastasis in nasopharyngeal carcinoma cells [1]. However, the mechanism of its function in cancer growth and metastasis is still unclear.

There were a few of reports on miR-1207's role in cancer biology. Chen L *et al.* showed that miR- 1207 suppresses gastric cancer growth and invasion by targeting telomerase reverse transcriptase (hTRET) [2]. MiR-1207-5p was significantly upregulated in gastric cancer patients without lymph node metastasis (LNM) compared with those with LNM [3]. Recently, Wu *et al.* discovered that miR-1207 directly targetes multiple negative regulators (SFRP1, AXIN2 and ICAT) of Wnt/β-catenin signaling and promoted cancer stem cell–like traits in ovarian cancer [4]. So, more target genes are needed to be identified for miR-1207-5p in a variety of cancer types.

In the current study we identified a novel target gene *CSF1* for miR-1207-5p, and investigated its role in tumorigenesis of lung cancer. Colony stimulating factor 1 (CSF1, also known as macrophage (M)-CSF) is a hemopoietic growth factor for the mononuclear phagocyte lineage and the primary regulator of macrophage differentiation, proliferation and survival. CSF1 elicits its effect through binding with CSF1 receptor (CSF1R) which is a high-affinity receptor tyrosine kinase encoded by the *c-fms* proto-oncogene. CSF1 is secreted by a variety of cell types and acts both locally and humorally in an autocrine and paracrine manner. CSF1 and CSF1R have been reported to be expressed by the tumor epithelium in several human epithelial cancers, including breast, ovarian, lung, endometrial, trophoblastic and prostatic cancer. [5–14] Under this circumstance, we suggested that mR-1207-5p plays an important role in tumor microenvironment through regulating *CSF1*.

## RESULTS

### miR-1207-5p directly targets and inhibits *CSF1*

**Figure 1:**
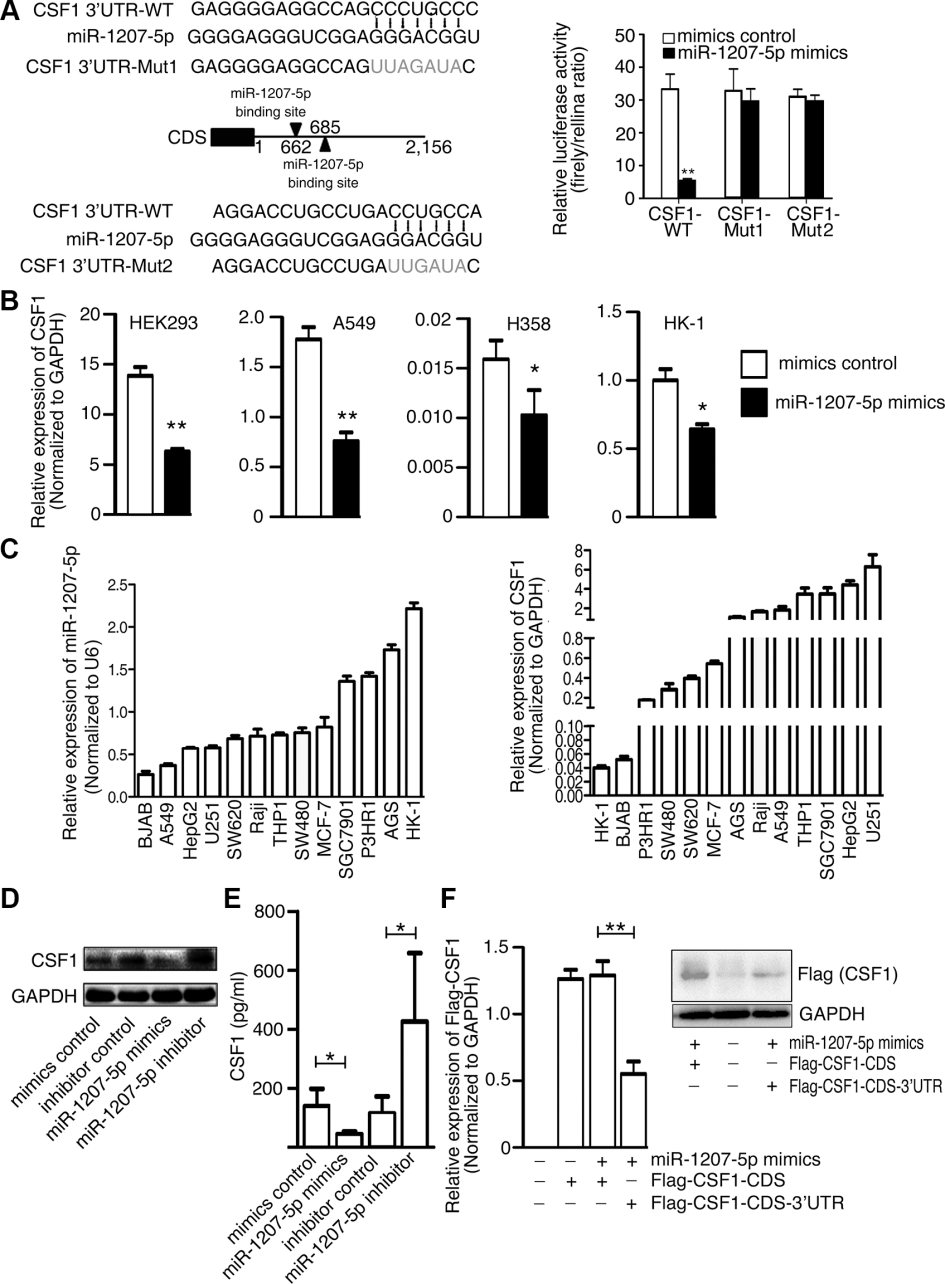
*CSF1* is a direct target of miR-1207-5p (**A**) Left: Scheme for the potential binding sites of miR-1207-5p in the 3′-UTR of *CSF1*, and the sequence of each intact miR-1207-5p binding site (wild-type, WT) and its mutant (Mut) within the luciferase reporter vector. Right: HEK-293 cells were cotransfected with the miR-1207-5p mimics (or control) and a luciferase reporter containing the 3′-UTR (WT or Mut) of CSF1 for 36 hrs. Cell lysates were then harvested for luciferase activities assay. (**B**) HEK293, A549, H358 and HK-1 cells were transfected with either miR-1207-5p mimics or mimics control for 48 hrs, the *CSF1* mRNA levels were then analyzed by RT–qPCR. (**C**) RT-qPCR analysis of the expression levels of *CSF1* and miR-1207-5p in multiple cancer cell lines. (**D–E**) A549 cells were transfected with either miR-1207-5p mimics or inhibitors for 48 hrs. CSF1 protein levels in cell lysates were analyzed by Western blotting (D), and in cell supernatant were analyzed by ELISA (E). (**F**) A549 cells were transfected with indicated vectors and mimics for 48 hrs. Flag-CSF1 mRNA and protein levels were analyzed by RT–qPCR (left) or Western blotting (right). All data are shown as means ± s.e.m. *P < 0.05, **P < 0.01 compared with control.

To explore the function of miR-1207-5p in the EMT and metastasis processes, three computational algorithms (TargetScan, PicTar and miRanda) were used to search for potential miR-1207-5p target genes. Among these candidate target genes, *CSF1*, which was predicted by all three algorithms, attracted our attention. CSF1 is a cytokine that controls the production, differentiation, and function of macrophage. CSF1 Promotes the release of proinflammatory chemokines, and thereby plays an important role in innate immunity and in inflammatory processes. CSF1 expression by macrophages, stromal cells and osteoclasts allows for important paracrine interactions between host microenvironment and cancer cells. Two miR- 1207-5p-binding sites were found in the 3′-UTR of *CSF1* mRNA, and there were perfect base pairing between the seed sequence of mature miR-1207-5p and the 3′-UTR of *CSF1* mRNA (Figure 1A left). We subcloned the full-length *CSF1* 3′-UTR into a luciferase reporter vector. Figure 1A (right) shows that miR-1207 mimics can inhibit the wild-type *CSF1* 3′-UTR luciferase activity, but the inhibition ability is compromised for two mutant *CSF1* 3′-UTR vectors. We transfected miR-1207-5p mimics into HEK293, A549, H358, and HK-1 cells, and found that miR- 1207-5p mimics reduced *CSF1* mRNA levels (Figure 1B).

Next, we used quantitative real-time PCR (qPCR) to measure miR-1207-5p and *CSF1* expression levels in different cancer cell lines. Figure 1C shows that lung adenocarcinoma cell line A549 has relative low miR- 1207-5p expression and high *CSF1* expression, so, we chose the A549 cell for the *in vitro* study. Western blotting and ELISA revealed that miR-1207-5p mimics reduced the CSF1 protein levels and its secretion, while miR-1207-5p inhibitor increased its levels in A549 cells (Figure 1D, 1E). We also transfected expression vector of *CSF1*-CDS (with or without 3′-UTR) to the A549 cells with miR-1207-5p mimics, respectively, and found that miR-1207-5p only can decrease *CSF1* expression when the expression-vector has *CSF1* 3′-UTR, which suggested the inhibition of *CSF1* expression by miR-1207-5p was dependent on *CSF1* 3′-UTR (Figure 1F). These results provided evidence that miR-1207-5p directly recognize the 3′-UTR of *CSF1*, and thereby inhibit their translation.

### miR-1207-5p inhibits lung cell proliferation and invasion *in vitro*

**Figure 2:**
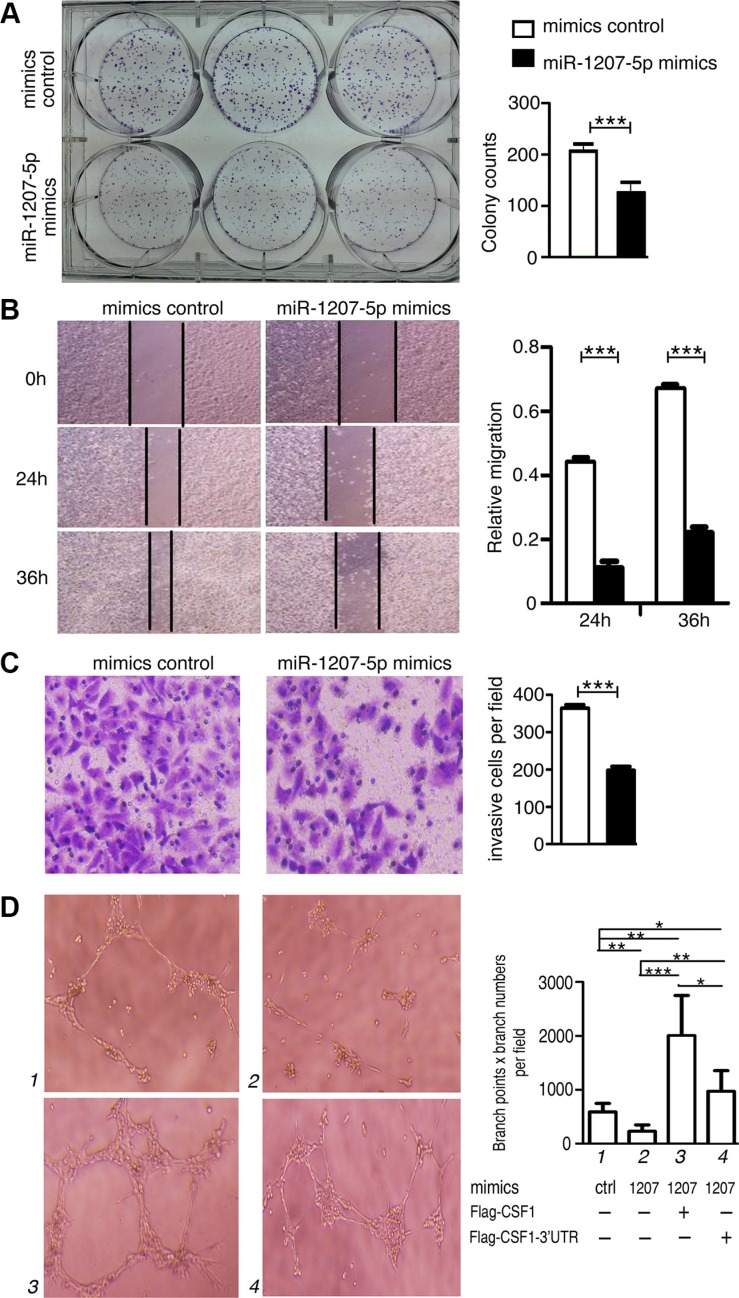
miR-1207-5p suppresses the tumorigenicity of A549 cells and inhibit tube formation of endothelial cells *in vitro* (**A**) A549 cells were transfected with either miR-1207-5p mimics or mimics control for 24 hrs, and 1000 cells were seeded into 6-well plates for 7 days to assay the cloning formation ability. (**B**) A549 cells were transfected with either miR-1207-5p mimics or mimics control for 24 hrs, and were seeded into 6-well plates to assay the wound-healing ability. (**C**) A549 cells were transfected with either miR- 1207-5p mimics or mimics control for 24 hrs, and were seeded into the insert of transwell to assay the invasion ability. Representative figures of the migrated stained cells are shown. The cells in five randomly selected areas were counted. (**D**) A549 cells were transfected with indicated mimics or vectors for 48 hrs, and then, the cell supernatants were collected and added to the medium of HUVEC to assay their effect on the tube formation ability of HUVEC. All data are shown as the mean ± s.e.m. *p < 0.05, **p < 0.01, ***p < 0.001 compared with control.

In light of the above findings, we decided to explore the biological significance of miR-1207-5p in lung tumorigenesis. We found that miR-1207-5p mimics markedly attenuated A549 cells proliferation compared to the control group through a colony formation assay (Figure 2A). We also noticed that miR-1207-5p mimics inhibited the *in vitro* migrative and invasive potentials of A549 cells compared to the control group (Figure 2B, 2C). We also observed similar effect of miR-1207-5p in another lung cancer cell line H358 (Supplementary Figure 1). We previously have revealed that miR-1207-5p can suppress a few of EMT-related molecules [1], and here, we also found that it can downregulate some important EMT molecules, such as *Snail*, *Smad2*, *Smad3*, Smad7, *Vimetin*, *ZEB1* in A549 cells (Supplementary Figure 2).

We next explored whether the miR-1207-5p mimics could affect angiogenesis using an endothelial cell tube formation assay. As shown in Figure 2D, medium from A549 cells overexpressing miR-1207-5p inhibited HUVEC cells tube formation compared to the control group. We also transfected construct encoding *CSF1*-CDS (with or without 3′-UTR) to the A549 cells with miR-1207-5p mimics, respectively, and found that *CSF1* without 3′-UTR, but not the *CSF1* with 3′UTR, can significantly increase the angiogenic potential of the cells under the influence of miR-1207-5p overexpression (Figure 2D). We also transfected the HUVEC cells with either miR-1207-5p mimics or control mimics, and found that miR-1207-5p can directly suppress the angiogenic ability of HUVEC cells (Supplementary Figure 3). These observations suggested that miR-1207-5p can suppress the proliferation, migration, and invasion of lung cancer cells *in vitro* and it also can inhibit endothelial cell tube formation to some extent.

### miR-1207-5p inhibits the AKT and STAT3 signaling

**Figure 3:**
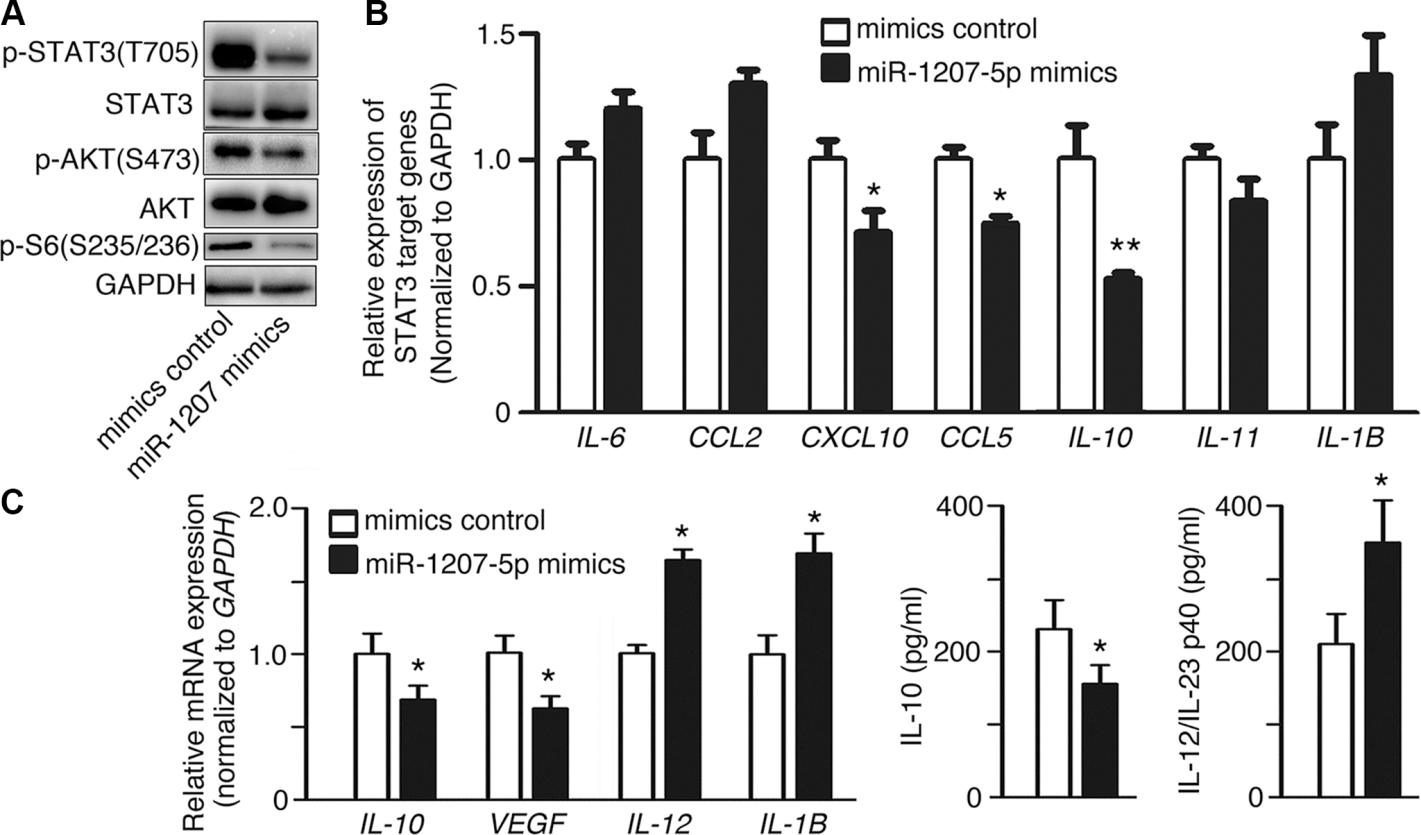
miR-1207-5p inhibits the AKT and STAT3 signalings (**A–B**) A549 cells were transfected with either miR-1207-5p mimics or control mimics for 48 hrs, and the cellular proteins and RNAs were isolated. The cellular protein levels of p-STAT3, p-AKT, p-S6 were assayed by Western blotting (A). The STAT3 signaling downstream target genes (*IL-6, CCL2, CXCL-10, CCL5, IL-10, IL-11, IL-1B*) mRNA expression levels were assayed by RT-qPCR (B). (**C**) To assay the effect of miR-1207-5p on macrophage characters, d-THP1 cells were transfected with either miR-1207-5p mimics or control mimics for 48 hrs, then, total RNA was isolated and RT-qPCR was performed to assay the M1/M2 macrophage phenotype-related genes expression levels (left); and IL-10 and IL-12/23 protein levels from cell supernatant were assayed by ELISA (right). All data are shown as the mean ± s.e.m. *p < 0.05, **p < 0.01 compared with control.

CSF1-CSF1R activation can trigger several important signaling pathways, such as the PI3K–AKT and STAT3 pathways [15–17], most of which regulate cell proliferation, invasion, and inflammation. Therefore, we investigated the possibility that miR- 1207-5p regulates those pathways by targeting *CSF1*. miR-1207- 5p mimics decreased the phosphorylation levels of AKT and AKT signaling downstream target S6 (Figure 3A). We also observed that miR-1207-5p mimics inhibited phosphorylation levels of STAT3 and its downstream target genes including *CXCL10*, *CCL5*, and *IL-10* (Figure 3A, 3B) [18, 19]. Macrophage plays vital role in the tumor microenvironment [20–22], and CSF1 is essential for proliferation, differentiation, and function of macrophage [6]. We next explored whether the miR- 1207-5p mimics could modulate macrophage's function. Macrophage cell d-THP1 was transfected with either miR-1207-5p mimics or control mimics, RT-qPCR of cellular RNA showed that miR-1207-5p can suppress *IL-10* and *VEGF* (M2 macrophage characters) expression levels, whereas increase *IL-12* and *IL-1B* (M1 macrophage characters) levels; ELISA of the supernatant also confirmed that miR-1207-5p inhibited IL-10 secretion, whereas promoted IL-12/-23 secretion (Figure 3C).

### miR-1207-5p suppresses lung cancer cell metastasis *in vivo*

**Figure 4:**
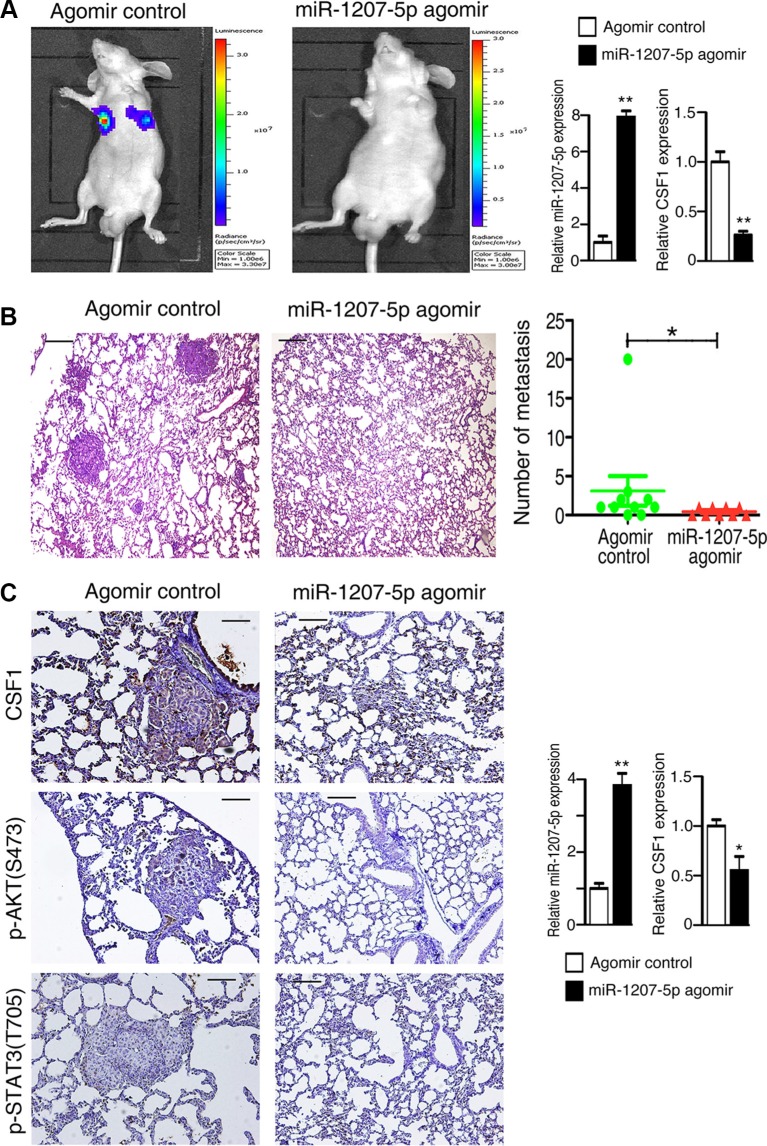
miR-1207-5p suppresses the metastasis of A549 cells in a nude mouse xenograft model The tail veins of nude mice were intravenously injected with A549-luciferase cells (which had been treated with either miR-1207-5p agomir or a control agomir for 24 hrs). miR-1207-5p agomir group has nine mice and control agomir group has ten mice. After 7 days, each group of nude mice were treated with miR-1207-5p agomir or control agomir through vein injection, respectively. Fifty days later, D-Luciferin was administered to each mouse, and the mice were then imaged by using a Xenogen IVIS Lumina II imaging system. After then, necropsies and pathology examination were performed. (**A**) Representative images of IVIS imaging, which revealed the metastatic nodules in the lung (left). Effects of miR- 1207-5p agomir on miR-1207-5p and CSF1 mRNA expression in A549 cells assayed by RT-qPCR (right). (**B**) Representative images of H&E– stained mice lung sections, which revealed the metastatic nodules (left), and numbers of metastases in lungs of individual mice were analyzed (right). (**C**) The expression of CSF1, p-AKT and p-STAT3 were assayed by immunohistochemistry (left). The mRNA levels of miR-1207-5p and CSF1 of mice lung tumor tissues were analyzed by RT-qPCR (right). Scale bar: 50 μm. All data are shown as the mean ± s.e.m. *p < 0.05, **p < 0.01 compared with control.

Because overexpression of miR-1207-5p could inhibit tumorigenesis *in vitro*, we next asked whether it could inhibit the metastatic potential of A549 cells *in vivo*. A549-luciferase cells that had been treated with miR- 1207-5p agomir or control agomir were injected into the tail vein of nude mice, and the efficiency of transfection was verified (Figure 4A right). Lung metastasis was then examined using the Xenogen IVIS imaging system. High luciferase activity was observed in the lung in the mice receiving the cells transfected with control agomir, whereas significantly reduced luciferase activity was observed in the miR-1207-5p agmoir group (Figure 4A left). The lungs with metastases were resected and processed for H&E staining, which showed significant decrease of metastases the lungs of in miR-1207-5p agomir group compared to the control group (Figure 4B). Further, immunohistochemical staining revealed that treated with the miR-1207-5p agomir, resulted in decreased expression of CSF1, phos-AKT, and phos-STAT3 in the lung tissues (Figure 4C left). RNA isolated from the lung tumor tissues was used for RT-qPCR assay, and it showed that the miR-1207-5p expression levels were significantly increased in the miR-1207-5p agomir treatment group, whereas CSF1 expression is decreased compared with control group (Figure 4C right). These results indicated that miR- 1207- 5p suppressed the metastasis of A549 cells to lung *in vivo*.

### miR-1207-5p and CSF1 expression levels are associated with clinicopathological parameters of NSCLC patients

**Figure 5:**
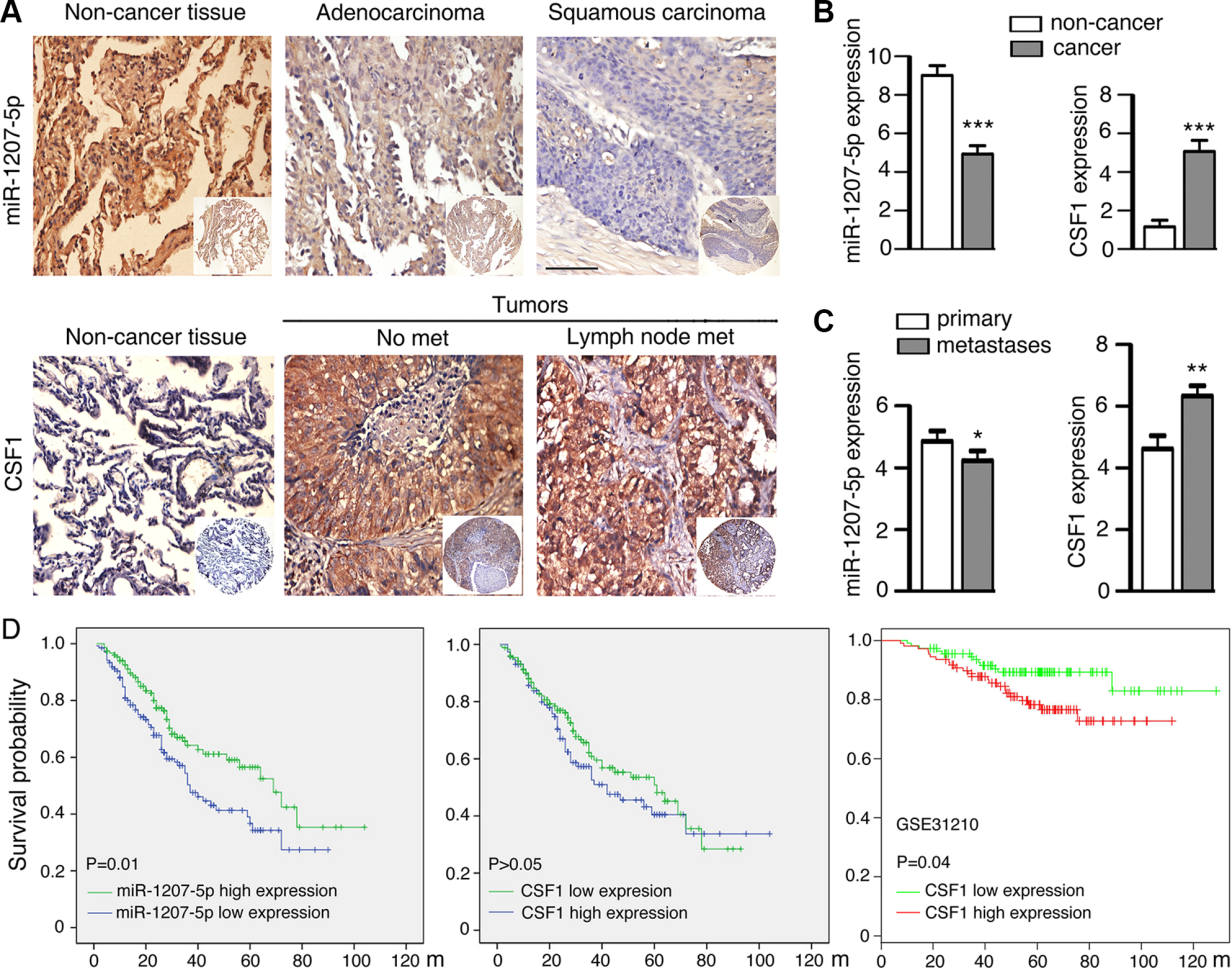
Expression of miR-1207-5p and CSF1 in NSCLC and control non-cancerous lung tissues by means of TMA (**A**) *In situ* hybridization staining of miR-1207-5p mRNA in adenocarcinoma and squamous carcinoma tissues as well as non-cancerous lung tissues (upper panel). Immunohistochemical staining of CSF1 proteins in lung cancer tissues (with or with out lymph node metastasis) and control non-cancerous lung tissues (bottom panel). Scale bar: 50 μm. (**B**) Expression scores of miR-1207-5p and CSF1 in lung cancer tissues (*n* = 289) and non-cancerous lung tissues (*n* = 53). (**C**) Expression scores of miR-1207-5p and CSF1 in lung cancer tissues, which had both primary tumor tissues and metastasis tumor tissues (*n* = 118 pairs). All data are shown as the mean ± s.e.m. *p < 0.05, **p < 0.01, ***P < 0.001 compared with control. (**D**) Kaplan-Meier analysis was used to plot the overall survival curves of 289 cases of NSCLC patients with different expression of miR-1207-5p and CSF1, which statistical significance was assessed by log-rank test (left and middle panel). MIRUMIR database [24] was used to analyze the clinic impact of CSF1 expression patterns on lung cancer patients' survival in a NSCLC cancer specimen expression profile dataset (GSE31210, *n* = 222) (right panel).

Lung cancer is a major cause of cancer-related mortality worldwide, and 80%–85% of lung cancer cases diagnosed with non-small cell lung cancer (NSCLC), among which adenocarcinoma (ADC) and squamous cell carcinoma (SCC) are the two major histological subtypes [23]. In this study, we examined miR-1207-5p mRNA expression levels and CSF1 protein expression levels by analyzing tissue microarray containing lung ADC (*n* = 151) and SCC (*n* = 138) specimens as well as non- cancerous lung tissues (*n* = 53). Strong positive expression of miR- 1207-5p was identified on cytoplasm of non-cancerous lung tissues, and much lower expression were detected in ADC and SCC. In contrast, strong positive expression of CSF1 was identified in the cytoplasm of cancer tissues, but only weak staining was observed in the non-cancerous lung tissues (Figure 5A, 5B). In this tissue microarray, there are 118 patients had primary cancer tissues as well as metastases tissues. We found that miR-1207-5p expression levels were decreased in metastases tissues relative to their primary tissues. In contrast, the CSF1 expression levels were higher in metastases compared with their primary tissues (Figure 5C).

In univariate survival analysis of NSCLC patients, Kaplan-Meier survival curve analysis with log-rank significance test was performed. The overall survival rates for NSCLC patients with high expression of miR- 1207-5p were significantly higher than these with low miR- 1207- 5p expression (*P* = 0.01, Figure 5D left). However, no significant association between expression of CSF1 and overall survival rates was noticed in this cohort patients (*P* > 0.05, Figure 5D middle). We next used MIRUMIR (a database for survival analyses in cancer [24]) and analyzed the clinic impact of *CSF1* expression patterns on lung cancer patients survival. In a NSCLC cancer specimen expression profile dataset (GSE31210, [25]), cancer with low expression of *CSF1* had better survival chance compared with cancer with high expression of *CSF1* (*P* = 0.04, Figure 5D right). The human NSCLC data supported the concept that miR-1207-5p has anti-metastasis or tumor suppressor functions, whereas CSF1 has pro-metastasis or oncogene function.

## DISCUSSION

In order to discovery new miRNAs involved in the EMT process, we previously used EGF or TGF-β-induced EMT cellular model, and identified that miR-1207-5p is induced by both EGF and TGF-β and functions as negative feedback regulator of EMT [1]. There are limited information on miR-1207-5p's role in tumorigenesis and metastasis. In this study, we identified a novel target gene *CSF1* for miR-1207-5p. miR-1207-5p can inhibit CSF1 mRNA/protein expression and secretion in lung cancer cell A549 by targeting the 3′-UTR of the *CSF1* mRNA.

As a growth factor, CSF1 is secreted by macrophages, epithelial and fibroblasts cells and cancer cells [26]. CSF1 promotes monocyte differentiating into macrophage, and support macrophage survival and proliferation. CSF1 and its receptor CSF1R play vital roles in normal development, and function as mediators of intercellular communication by diffusible molecules [27]. CSF1 can stimulate cancer cells directly, and it also can promote tumorigenesis indirectly through mobilizing and adjusting the host immune system responding to cancer cells. Tumor associated macrophages (TAMs) stimulated by CSF1 have a wide range of activities like promoting tumor growth, angiogenesis, extracellular matrix breakdown. High microvessel density has a stronger association with expression of CSF1 [28, 29], suggesting that CSF1 may mediate a pro-angiogenesis phenotype. CSF1 promotes the proliferation of TAMs and supports it to produce a few of pro-angiogenic factors including VEGF, bFGF, TNF-α, and others [30–32]. The TAM and those factors are important parts of the tumor microenvironment, regulating cancer development, which is called the M2 phenotype of macrophage (usually expressing high levels of IL-10, VEGF and Arg-1; whereas expressing low levels of IL-12, IL-23 and iNOS) [20, 21]. CSF1 related TAM secrets multiple cytokines and promote nonresolving inflammation, and it also degrades the ECM by secreting matrix-bound growth factors [33–35]. CSF1 of the cancer cells can increase the migration ability of macrophages through autocrine loop, thus promotes the tumor invasion and metastasis, and further stimulates the secretion of CSF1 [36].

We reported that miR-1207-5p can suppress lung cancer cell A540 proliferation, migration and invasion. We also discovered that miR-1207-5p can inhibit HUVEC tube formation, which is dependent on the *CSF1* 3′-UTR existing. Since STAT3 and AKT are two important signaling pathways downstream of CSF1/CSF1R, we assayed the effect of miR-1207-5p on these two pathways. It showed that miR-1207-5p can downregulate STAT3 and AKT signalings, as well as their downstream targets, including some important inflammatory mediators such as *IL-10*, *CCL5* and *CXCL10*. We also showed that miR-1207-5p can regulate a few of important EMT-related molecules such as Snail, Smad 2, Smad3, Smad7, Vimentin, and ZEB1, which indicated the inhibition role of miR-1207-5p in tumor invasion and metastasis.

We evaluated the effect of miR-1207-5p on macrophage function in d-THP1 cells. miR-1207- 5p mimics significantly reduced the M2 macrophage characters (such as IL-10 and VEGF), whereas increased the M1 macrophage characters (such as IL-12 and IL- 23). These results provide evidence that miR-1207- 5p could modulate macrophage function of the tumor microenvironment.

We also used nude mouse xenograft model to confirm that miR-1207-5p could suppress lung cancer cells metastasis *in vivo*. Immunohistochemistry staining showed that miR-1207-5p suppressed CSF1, phos-AKT, and phos-STAT3 expression in the mouse model.

At last, we found that miR-1207-5p expression levels were significantly downregulated in NSCLC specimens, whereas CSF1 expression levels were upregulated in NSCLC compared to the non-cancerous lung tissues. Also, miR-1207-5p expression levels were lower in metastases compared to their primary tissues, whereas CSF1 had the opposite effect. More importantly, high expression of miR-1207-5p or low expression of CSF1 provided better survival chance for NSCLC patients compared with cancer with low expression of miR-1207-5p or high expression of CSF1 (Figure 5D left and right). These clinic data implies the miR-1207-5p-CSF1 axis may play roles in the NSCLC development *in vivo*.

Tumor microenvironment provides multiple supports for tumor development and metastasis [34]. Our present study provides new information that miR-1207-5p can target *CSF1*, an essential growth factor for macrophage, and thus modulate the tumor microenvironment. miR-1207- 5p is an anti-metastasis microRNA, and understanding the miR- 1207-5p-CSF1 axis will provide additional opportunities for the design of treatments for NSCLC. The exact role of miR-1207-5p in carcinogenesis is worthy of further study.

## MATERIALS AND METHODS

### Vectors construction

The 3′-UTR of *CSF1* was amplified and subcloned into the pMIR-Report luciferase vector (Ambion, Austin, TX, USA). Mutants of the seed region of the putative miRNA-binding sites in 3′-UTR were generated by point mutation PCR. The CDS and CDS plus 3′UTR of *CSF1* were obtained by PCR from *CSF1* plasmid (Origene, Rockville, MD, USA) and subcloned into the pCMV-N-Flag vector (Beyotime Biotechnology, Jiangsu, China).

### Cell culture

HEK293, A549, H358, HK-1, BJAB, Raji, P3HR1, THP1, HepG2, U251, SW620, SW480, MCF-7, SGC7901 and AGS cells were grown in RPMI 1640 medium supplemented with 10% feal calf serum (FBS) at 37°C with 5% CO_2_. A549 and H358 are human NSCLC cell lines, and HK-1 is a human nasopharyngeal carcinoma cell line. BJAB, Raji and P3HR1 are B cell lymphoma cell lines, and HepG2 is a hepatocellular carcinoma cell line. U251 is a brain glioblastoma cell line, and MCF-7 is a breast cancer cell line. SGC7901 and AGS are gastric cancer cell lines, and SW480 and SW620 are colon cancer cell lines.

To obtain macrophage-like differentiated cells (d-THP1), human monocytic cell line THP-1 cells were treated with 25 ng/ml phorbol myristate acetate in the medium for 48 h. After the incubation, the phorbol myristate acetate-containing medium was aspirated and adherent differentiated cells were resuspended in fresh medium and incubated for an additional 24 h.

To assay the effect of miR-1207-5p on macrophage characters, d-THP1 cells were transfected with either miR-1207-5p mimics or control mimics for 48 h. Total RNA was purified using Trizol (Life Technologies, Gaithersburg, MD, USA) and RT-qPCR was performed to assay the M1/M2 macrophage phenotype-related genes expression levels.

### Quantitative real-time RT-PCR

Quantitative real-time RT-PCR was carried out as described previously [1]. The primer sequences are listed in the Supplementary Table 1.

### Luciferase activity assay

Luciferase activity assay was carried out as described previously [1].

### Wound-healing assay

A549 or H358 cells were transfected with a synthetic microRNA mimics (GenePharma, Shanghai, China) and then grown in a 6-well plate. After the cell monolayer had reached 90% confluence, a wound was made with a 10- μl pipette tip. Cells were then cultured in medium with 1% FBS, and migration at the corresponding wound site was documented using a microscope at the indicated time points.

### Matrigel invasion assay

Before cell seeding, 24-well Transwell plates (8- μm pores; Corning, New York, NY, USA) were coated with Matrigel matrix (BD Biosciences, San Diego, CA, USA). 5 × 10^4^ transfected A549 or H358 cells suspended in 200 μl volume of RMPI 1640 (with 1% FBS) were added to the top of each insert well. Medium with 15% FBS was placed in the bottom wells. The cells were then allowed to migrate for 48 h at 37°C. The invasived cells were fixed with 10% methanol for 15 minutes. Then the invasive cells on the lower surface of the membrane were stained with 2% crystal violet for 5 min, and the stained cells were counted under a microscope. To minimize bias, at least five fields with 100× magnification were counted, and the various counts were averaged.

### Colony formation assay

A549 or H358 cells were transfected with miRNA mimics and then grown in the 6-well plates for 24 h. For each group, 1000 cells were seeded in triplicate into 6-well plates for 7 days during which period the medium was not changed. Cells were then fixed with 4% formaldehyde and stained with crystal violet for 5 minutes and washed with PBS for three times.

### Tube formation assay

The tube formation assay was carried out as described [37]. In brief, 96-well plates were coated with 50 μl Matrigel. 2 × 10^4^ HUVEC cells were added to each well and incubated at 37°C for 6–8 h. Images were acquired under an inverted microscope. Antiangiogenic activity was quantified by measuring the length of tube walls formed between discrete endothelial cells in each well relative to the control.

### Western blot analysis

Western blotting was carried out as described previously [38]. Antibodies against phos-STAT3(T705) and phos-S6(S235/236) were obtained from Cell Signaling Technology (Danvers, MA, USA). Anti-CSF1, AKT, and phos-AKT (S473) antibodies were from Abcam (Cambridge, MA, USA). Anti-STAT3 antibody was from Abzoom (Dallas, TX, USA). Anti-Flag antibody was from Sigma–Aldrich (St. Louis, MO, USA). Anti-GAPDH antibody was from Millipore (Billerica, MA, USA).

### ELISA

Secretion of CSF1 was detected according to the ELISA Kit for Colony Stimulating Factor 1 assay manual (Cloud-Clone Corp, Houston, USA) after miR-1207-5p mimics or inhibitors were transfected into A549 cells for 48 h. Secretion of IL-10, IL-12/IL-23 was detected by ELISA Kits (purchased from R&D, Minneapolis, MN, USA) after miR-1207-5p mimics or control mimics were transfected into d-THP1 cells for 48 h.

### Animal experiment

A549-luciferase cells were harvested after treatment of miR-1207-5p agomir or control agomir (Ribobio, Guangzhou, China) for 24h, and single cell suspensions of 8×105 cells were used for vein injections of four-week-old male athymic BALB/c nude mice. miR-1207-5p agomir group has nine mice and control agomir group has ten mice. Seven days later, each group of nude mice were treated with miR-1207-5p agomir or control agomir (150 μl, 200 nM) through vein injection, respectively. Fifty days later, D-Luciferin was administered to each mouse by intraperitoneal injection at a dose of 150 mg/ kg, and the mice were anesthetized for 5 min in a chamber with 3% isofluorane. The mice were then imaged by using a Xenogen IVIS Lumina II imaging system (Caliper Life Sciences, Hopkinton, MA, USA). After then, necropsies were performed. After physical separation of the lobes of each lung, the metastases numbers on the surface of each of the five lobes were counted under a dissecting microscope. Tumor cells in lung metastases in individual mice were analyzed by immunohistochemistry. All animal procedures were performed in accordance with institutional guidelines. Agomir is chemically-modified double-strand miRNA mimics which can mimic mature endogenous miRNAs after transfection into cells.

### Tissue microarray (TMA) and clinical data

TMAs were constructed as described previously [39]. Samples were obtained with informed consent and all protocols were approved by The Second Xiangya Hospital of Central South University Ethics Review Board. Written informed consent was obtained from all patients. There are 289 cases of NSCLC patient lung cancer tissues (See Table 1), and 53 non-cancerous lung tissues in the TMA. Among the 289 cancer samples, 118 samples had both primary tumor tissues and metastasis tumor tissues.

**Table 1:** 289 cases of non small lung cancer (NSCLC) patient's characteristics

Patients characteristics	No. of cases (%)
**Age (years)**	
≤ 50	75 (26%)
> 50	214 (74%)
**Gender**	
Male	217 (75%)
Female	72 (25%)
**Clinical stages**	
I	75 (26%)
II	69 (24%)
III	134 (46%)
IV	11 (4%)
**LNM status**	
LNM	184 (64%)
No LNM	105 (36%)
**Histological type**	
SCC	138 (48%)
ADC	151 (52%)
**Histological grades**	
Well differentiation	5 (2%)
Moderate differentiation	125 (43%)
Poor differentiation	159 (55%)

Abbreviations: LNM, lymph node metastasis; SCC, squamous cell carcinoma; ADC, adenocarcinoma.

### Immunohistochemistry and scores

Immunohistochemistry staining and scores were carried out as described previously [39].

### In situ hybridization

*In situ* hybridization was carried out as describied prebiously [40]. 5′DIG-labeled has-miR-1207-5p miRCURY LNA detection probe was obtained from Exiqon (Vedbaek, Denmark).

### Statistical analysis

Statistical analysis was performed using SPSS17.0 and Graph-Pad Prism 5. Chi-square test or Kruskal–Wallis *H* test was used for categorical variables. Kaplan-Meier analysis was performed for overall survival curves and statistical significance was assessed using the log-rank test. Independent *t*-test or ANOVA was performed for qRT-PCR and phenotype analyses. Significance parameters were set at *p* < 0.05.

## SUPPLEMENTARY MATERIAL FIGURES AND TABLE


