# Supplementary material for: miR-1207-5p suppresses lung cancer growth and metastasis by targeting CSF1
Source: Oncotarget. 2016 Apr 13;7(22):32421–32. doi: 10.18632/oncotarget.8718 (PMC5078023; doi:10.18632/oncotarget.8718)
Supplement: Supplementary file 1 [file oncotarget-07-32421-s001.pdf]

miR-1207-5p suppresses lung cancer growth and metastasis by targeting CSF1

Supplementary Materials

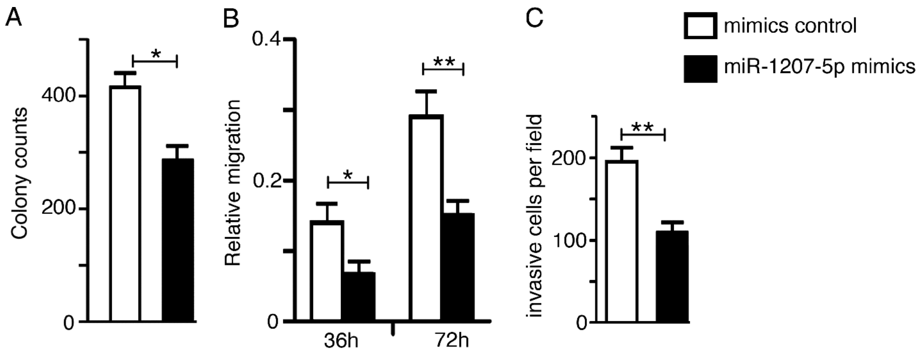

Supplementary Figure S1: miR-1207-5p suppresses the tumorigenicity of H358 cells *in vitro*. (**A**) H358 cells were transfected with either miR-1207-5p mimics or mimics control for 24 hrs, and 1000 cells were seeded into 6-well plates for 7 days to assay the cloning formation ability. (**B**) H358 cells were transfected with either miR-1207-5p mimics or mimics control for 24 hrs, and were seeded into 6-well plates to assay the wound-healing ability after 36 hrs and 72 hrs. (**C**) H358 cells were transfected with either miR-1207-5p mimics or mimics control for 24 hrs, and were seeded into the insert of transwell to assay the invasion ability. All data are shown as the mean ± s.e.m. *$p < 0.05$, **$p < 0.01$ compared with control.

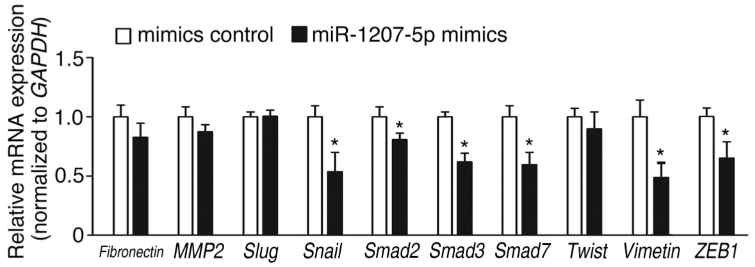

Supplementary Figure S2: miR-1207-5p regulated important EMT-related genes. A549 cells were transfected with either miR-1207-5p mimics or control mimics for 48 hrs, and the cellular RNAs were isolated. EMT related genes expression levels were assayed by RT-qPCR. Data are shown as the mean ± s.e.m. *$p < 0.05$ compared with control.

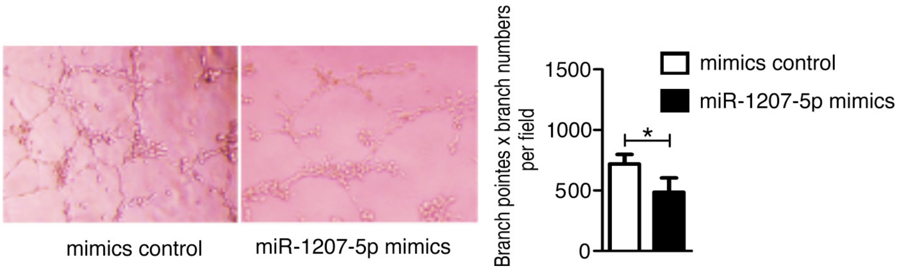

Supplementary Figure S3: miR-1207-5p inhibited HUVEC cells tube formation abilities. HUVEC cells were transfected with either miR-1207-5p mimics or control mimics for 48 hrs, then, the cells were processed to tube formation ability assay. Data are shown as the mean ± s.e.m. *$p < 0.05$ compared with control.

Supplementary Table S1: Primers list for qPCR

CCL2 F	GACCATTGTGGCCAAGGAGA
CCL2 R	TTGGGTTTGCTTGTCCAGGT
CCL5 F	CCAGCAGTCGTCTTTGTCAC
CCL5 R	CTCTGGGTTGGCACACACTT
CXCL10 F	TGGCATTCAAGGAGTACCTCTC
CXCL10 R	GGACAAAAT TGGCTT GCAGGA
CSF1 F	TGCGTCCGAACTTTCTATG
CSF1 R	CACTGCTAGGGATGGCTTT
Fibronectin F	GGTGACACTTATGAGCGTCCTAAA
Fibronectin R	AACATGTAACCACCAGTCTCATGTG
Flag-CSF1 F	TACAAGGATGAC GAC GATAAGAGC
Flag-CSF1 R	AGGT AGCACACT GGAT CTTT CA
GAPDH F	GAAGGT GAAGGT C GGAGT C
GAPDH R	GAAGAT GGT GAT GGGATTT C
IL-1B F	ATGATGGCTTATTACAGTGGCAA
IL-1B R	GT CGGAGATT CGTAGCTGGA
IL-6 F	GGTACATCCTCGACGGCATCT
IL-6 R	GTGCCTCTTTGCTGCTTTCAC
IL10 F	CGAGATGCCTTCAGCAGAGT
IL10 R	AATCGATGACAGCGCCGTAG
IL-11 F	CGAGCGGACCTACTGTCCTA
IL-11 R	GCCCAGTCAAGTGTCAGGTG
IL12B F	AGGGACAT CAT CAAAC C T GAC C
IL12B R	GCTGAGGTCTTGTCCGTGAA
MMP2 F	CAACTACGAT GAT GACCGCAA
MMP2 R	GT GTAAAT GGGT GCCAT CAGG
SMAD2 F	CGTCCATCTTGCCATTCACG
SMAD2 R	CTCAAGCTCATCTAATCGTCCTG
SMAD3 F	TTAGGTCACTGCTGGGCTGA
SMAD3 R	CCATCCAGGGACTCAAACGTG
SAMD7 F	TGCTGTGCAAAGTGTTCAGG
SMAD7 R	GCATCT GGACAGT CAGTTGGT
SNAI1 F	GCTGCAGGACTCTAATCCAGAGTT
SNAI1 R	GACAGAGTCCCAGAT GAGCATTG
SNAI2 F	AGATGCATATTCGGACCCAC
SNAI2 R	CCTCATGTTTGTGCAGGAGA
Twist1 F	CAGCGCACCCAGT CGCT GAA
Twist1 R	CCAGGCCCCCTCCATCCTCC
U6 F	ATT GGAAC GAT ACAGAGAAGAT T
U6 R	GGAACGCTTCACGAATTTG
VEGF F	CTTGCCTTGCTGCTCTACCT
VEGF R	GCAGTAGCTGCGCTGATAGA
Vimentin F	AGATGGCCCTTGACATTGAG
Vimentin R	TGGAAGAGGCAGAGAAATCC
ZEB1 F	GCACAAC CAAGT GCAGAAGA
ZEB1 R	GCCTGGTT CAGGAGAAGAT G
